# Diagnostic value of platelet-lymphocyte ratio for pediatric sepsis: A systematic review and meta-analysis

**DOI:** 10.12669/pjms.42.8.19637

**Published:** 2026-08

**Authors:** Li Chen, Qiuping Yang

**Affiliations:** 1 Li Chen Department of Neonatology, Huzhou Maternity & Child Health Care Hospital, Huzhou, Zhejiang Province 313000, P.R. China; 2 Qiuping Yang Department of Pediatric, Huzhou Maternity & Child Health Care Hospital, Huzhou, Zhejiang Province 313000, P.R. China

**Keywords:** Biomarker, Diagnosis, Inflammation, Sepsis

## Abstract

**Objectives:**

Early diagnosis of pediatric sepsis remains challenging due to limitations of conventional biomarkers. The platelet-to-lymphocyte ratio (PLR), derived from routine blood tests, has emerged as a potential diagnostic marker. This study aimed to evaluate the diagnostic accuracy of PLR for pediatric sepsis.

**Methodology:**

A systematic search of PubMed, Embase, Web of Science, and Scopus was conducted from inception to March 15, 2026. Studies assessing the diagnostic performance of PLR in pediatric sepsis and reporting sufficient data to construct 2×2 contingency tables were included. Pooled sensitivity and specificity were calculated using a random-effects model. Summary receiver operating characteristic (SROC) analysis and Deeks’ funnel plot were performed.

**Results:**

Fifteen studies with 1,980 patients were included. The pooled sensitivity and specificity of PLR for diagnosing pediatric sepsis were 0.81 (95% CI: 0.73–0.87) and 0.72 (95% CI: 0.60–0.82), respectively. The SROC curve demonstrated an area under the curve of 0.79, indicating moderate diagnostic accuracy. Significant heterogeneity was observed. Subgroup analysis showed better performance in larger studies (≥150 patients) and studies using higher PLR cut-offs (>50). Deeks’ funnel plot showed no evidence of publication bias (p = 0.536).

**Conclusions:**

Evidence from studies with high-risk of bias indicates that PLR may have moderate diagnostic accuracy for pediatric sepsis and may serve as a simple, cost-effective adjunctive biomarker. Since, most studies were on neonatal population, evidence may not be generalized to older pediatric populations. Further large-scale prospective studies are needed to establish standardized cut-off values and validate its clinical utility.

**Registration No:** PROSPERO (No. CRD420261338676).

## INTRODUCTION

Sepsis is a life-threatening condition caused by an abnormal immune response to infection, leading to organ failure and, if untreated, death.^1^ Neonates and children are disproportionately affected by this condition. The 2017 Global Burden of Disease study estimated about 20.3 million new cases of sepsis annually in children under 5, and 4.9 million cases in those aged 5–19.^2^ A systematic review by Fleischmann-Struzek et al. found a global neonatal sepsis rate of 2,202 per 100,000 live births (95% CI: 1,099–4,360), with higher incidence in South Asia and sub-Saharan Africa.^3^ Pediatric sepsis has serious mortality risks, with rates generally between 1–5%, increasing to 9–20% in severe cases.^4^ Survivors also face long-term effects termed post-sepsis syndrome, which includes neurodevelopmental issues, ongoing disability, and lower quality of life.^5^,^6^ These facts highlight that the need for swift, precise diagnosis of sepsis remains a critical area of research.

Traditionally, blood culture has been the gold standard for sepsis diagnosis. However, it is not ideal in clinical practice because results take 48–72 hours and are positive in only 40–60% of cases, leaving clinicians to make important treatment decisions without clear diagnostic confirmation.^7^ In this context, various biochemical and haematological biomarkers have been added to clinical practice. Currently, C-reactive protein (CRP) and procalcitonin (PCT) are the most common serum markers used in pediatric and neonatal sepsis evaluations.^7^-^9^ PCT, which increases within 3–4 hours of bacterial infection and decreases quickly with effective treatment, has generally been more sensitive and faster-responding than CRP.^8^ Additionally, markers such as interleukin-6 (IL-6), presepsin, CD64 on neutrophils, and soluble triggering receptor expressed on myeloid cells-1 have been studied, with varying levels of clinical potential.^10^ Nevertheless, these markers have limitations pertaining to cost, availability, and the need for specialised assay platforms.

In this context, interest has grown in haematological indices derived from routine blood tests as simple, accessible, and cost-effective indicators of systemic inflammation.^10^,^11^ The platelet-to-lymphocyte ratio (PLR), in particular, has been actively researched as it reflects two key aspects of immune dysregulation in sepsis. During sepsis, platelets are activated by vascular endothelial damage, forming aggregates with leukocytes that exacerbate inflammation and cause thrombocytosis; at the same time, lymphocytes undergo apoptosis and are sequestered in lymphoid tissues in response to cytokine storms, leading to lymphocytopenia.^12^,^13^ These opposing changes, increasing platelets and decreasing lymphocytes, likely enhance the PLR in septic patients, making it a possible inflammatory marker.

This is particularly beneficial for nursing personnel, as they are typically the first responders to detect early signs of changes in a child’s condition and are accountable for prompt laboratory monitoring and intervention in case of disease escalation. Integrating PLR into the early warning system can enhance nurse-led assessments of illness and facilitate the rapid identification of sepsis. While some studies have explored PLR for diagnosing pediatric sepsis, results have been inconsistent. A previous meta-analysis included only six studies, limiting definitive conclusions.^11^ Therefore, this systematic review and meta-analysis aimed to evaluate the diagnostic value of PLR for pediatric sepsis.

## METHODOLOGY

Two reviewers independently conducted a detailed literature search encompassing the electronic databases of PubMed, Embase, Web of Science, and Scopus. Additionally, Google Scholar was explored as the source of gray literature. The search included all records from the start of each database up to March 15, 2026. There were no language restrictions applied. A search algorithm was generated with the help of an experienced Medical Librarian while combining MeSH terms and free-text keywords. Details are provided in Supplementary Table-I. Search strategies for other databases were adjusted accordingly, using specific controlled vocabularies such as Emtree terms in Embase. Additionally, reference lists of all full-text articles and relevant systematic reviews were manually examined to find studies missed during the electronic search. The review was reported following the PRISMA guidelines^14^ and registered on PROSPERO (No. CRD420261338676).

**Supplementary Table-I:** Search strategy.

***PubMed***
(“Sepsis”[Mesh] OR sepsis OR septicemia OR “septic shock” OR “bloodstream infection” OR bacteremia) AND (“Platelet-Lymphocyte Ratio” OR “platelet lymphocyte ratio” OR “platelet-to-lymphocyte ratio” OR PLR OR “platelet lymphocyte count ratio”) AND (“Infant”[Mesh] OR “Child”[Mesh] OR “Adolescent”[Mesh] OR pediatric OR paediatric OR neonate OR newborn OR infant OR child OR adolescent).
***Embase***
(‘sepsis’/exp OR sepsis OR septicemia OR ‘septic shock’ OR ‘bloodstream infection’ OR bacteremia) AND (‘platelet lymphocyte ratio’ OR ‘platelet-to-lymphocyte ratio’ OR PLR OR ‘platelet lymphocyte count ratio’) AND (‘child’/exp OR ‘infant’/exp OR ‘adolescent’/exp OR pediatric OR paediatric OR neonate OR newborn OR infant OR child OR adolescent).
***Web of Science***
TS = ((sepsis OR septicemia OR “septic shock” OR “bloodstream infection” OR bacteremia) AND (“platelet lymphocyte ratio” OR “platelet-to-lymphocyte ratio” OR PLR OR “platelet lymphocyte count ratio”) AND (pediatric OR paediatric OR neonate OR newborn OR infant OR child OR adolescent))
Scopus.
TITLE-ABS-KEY ((sepsis OR septicemia OR “septic shock” OR “bloodstream infection” OR bacteremia) AND (“platelet lymphocyte ratio” OR “platelet-to-lymphocyte ratio” OR PLR OR “platelet lymphocyte count ratio”) AND (pediatric OR paediatric OR neonate OR newborn OR infant OR child OR adolescent))

### Inclusion Criteria:

- Evaluated the diagnostic performance of PLR for the detection of sepsis in a pediatric population (age ≤0–18 years).
- Reported sufficient data to reconstruct or extract the 2×2 contingency table or reported sensitivity and specificity with sufficient information to derive these values.
- Were of retrospective cohort, prospective cohort, or cross-sectional diagnostic accuracy design.
- Used a clinical or microbiological reference standard for the diagnosis of pediatric sepsis.
- ***Exclusion criteria:***
- Case reports, editorials, letters, conference abstracts.
- Diagnostic accuracy data could not be extracted.
- Duplicate publications using the same patient cohort.
- Studies that used PLR in combination with other biomarker.

### Study Selection and Data Extraction:

All search records were downloaded into EndNote software for electronic deduplication. Study selection was then conducted independently by two reviewers. First, they screened titles and abstracts of all retrieved records against predefined eligibility criteria. Next, full-text articles of studies that appeared potentially eligible were obtained and evaluated for final inclusion. Any disagreements at each stage were resolved through discussion between the reviewers.

Data extraction was also performed independently by two reviewers to extract the following information from the studies: first author’s name, publication year and country, study design, total sample size, number of sepsis cases and non-septic controls, age details of the study population, PLR diagnostic cut-off value, data for 2×2 contingency table (TP, FP, FN, TN), reported sensitivity and specificity, and the area under the receiver operating characteristic curve (AUC) where available. Any discrepancies in the data were resolved by cross-checking against the original reports and discussions among the reviewers.

### Quality assessment:

Two independent reviewers assessed the methodological quality and risk of bias of each study using the Quality Assessment of Diagnostic Accuracy Studies-2 (QUADAS-2) tool.^15^ This tool examines four domains:

- Patient selection.
- The index test.
- The reference standard.
- Flow and timing.

Each domain was rated for bias risk, with the first three also evaluated for applicability concerns. Ratings were categorised as “low risk,” “moderate risk,” or “high risk” for bias, and as “low concern,” “moderate concern,” or “high concern” for applicability, based on specific signalling questions relevant to diagnostic accuracy. Any disagreements between reviewers were resolved through discussion.

### Statistical analysis:

All statistical analyses were performed using Python (version 3.12) with the *NumPy*, *SciPy*, *pandas*, and *matplotlib* libraries. Pooled sensitivity and specificity were estimated using the DerSimonian–Laird random-effects model applied to logit-transformed values. A zero-cell continuity correction of +0.5 was applied to all four cells of the 2×2 contingency table for any study in which at least one cell count equaled zero, in order to prevent undefined logit transformations and to stabilise within-study variance estimates. Sensitivity and specificity data were combined to generate pooled effect estimates with 95% confidence intervals (CI). Heterogeneity was categorized as low (I² < 25%), moderate (I² 25–50%), substantial (I² 50–75%), or considerable (I² > 75%).^3^ The overall summary receiver operating characteristic (SROC) curve was constructed using the Moses–Littenberg method. Publication bias was assessed using Deeks’ funnel plot. We also conducted subgroup analyses based on country of origin, sample size, and PLR cut-off to explore potential sources of inter-study heterogeneity for both sensitivity and specificity.

## RESULTS

**Fig.1:**
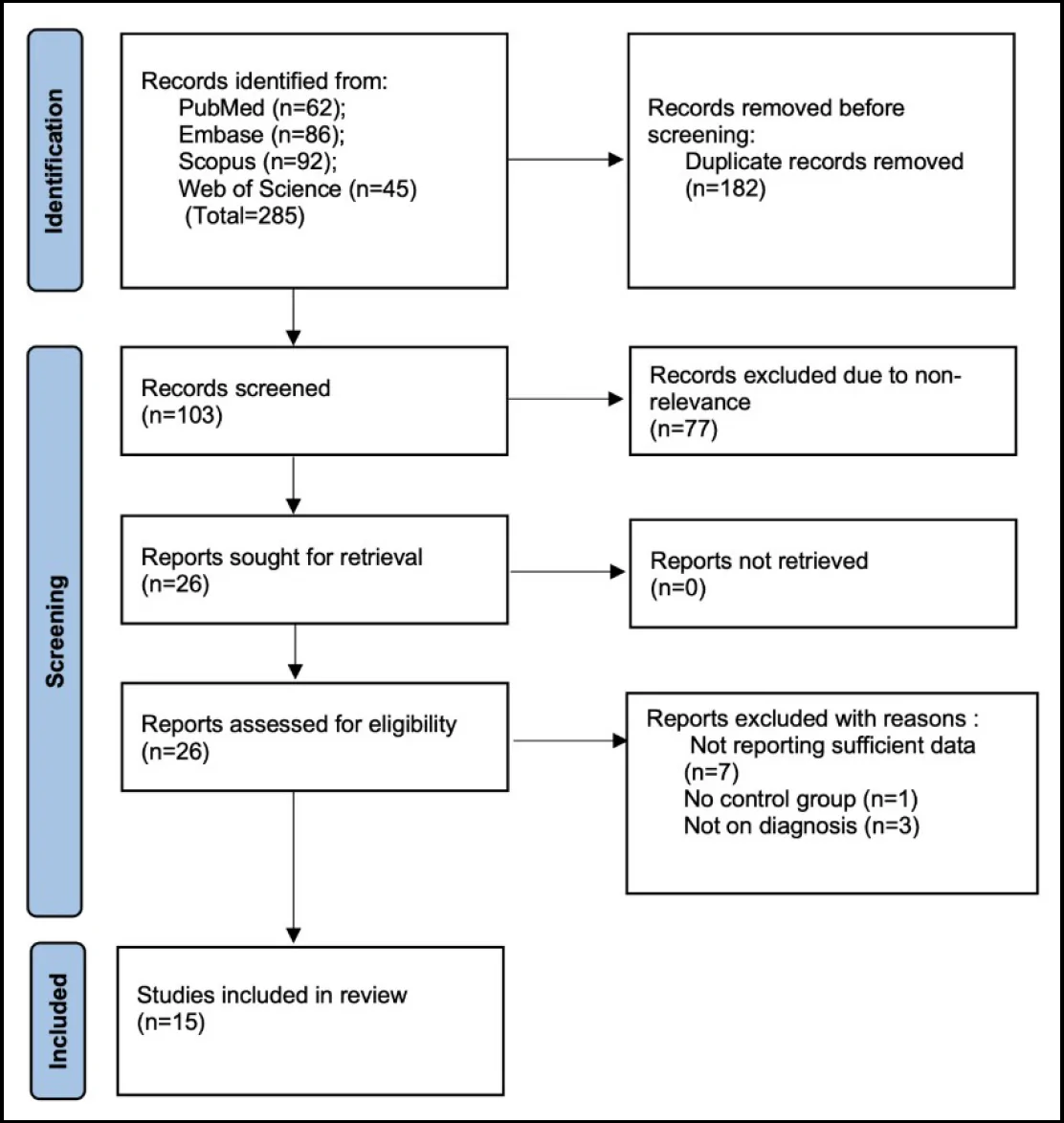
Study flowchart.

The study flowchart is shown in Fig.1. After a systematic search of the database, 1,980 patients from 15 eligible studies^12,13,16-28^ published between 2017 and 2025 were identified and included in the final meta-analysis. Most studies were retrospective. The studies originated from four countries: China, Egypt, Turkey, and Indonesia. Among the patients, 1,012 (51.1%) were diagnosed with sepsis, while 968 (48.9%) formed the non-septic control group. Most studies included neonatal populations. The reference standard used was clinical diagnosis with or without culture across the majority of studies (Table-I).

**Table-I:** Baseline details of included studies.

Study	Country	Study design	Sepsis type	Sample size	Sepsis	Controls	PLR Cut-off	Sensitivity	Specificity	AUC	Reference standard
Omran 2018	Egypt	P	EOS+LOS	70	35	35	73.10	0.514	0.457	0.460	Blood culture + clinical criteria
Can 2017	Turkey	P	EOS	122	78	44	94.05	0.974	1.000	0.930	Positive blood/CSF culture + clinical criteria
Arcagok 2019	Turkey	R	EOS	159	67	92	57.70	0.910	0.978	0.847	Blood culture + clinical criteria
Nasser 2020	Egypt	R	EOS	60	30	30	47.40	0.900	1.000	NR	Blood culture + clinical criteria
Zhang 2021	China	R	EOS	124	74	50	90.85	0.649	0.800	0.699	Blood culture
Kurt 2022	Turkey	R	EOS+LOS	97	20	77	37.72	0.800	0.078	0.268	Blood culture
Mahmoud 2022	Egypt	P	EOS	160	80	80	7.00	0.700	0.725	0.780	Blood culture + clinical criteria
Soliman 2022	Egypt	R	EOS	50	30	20	0.02	0.833	0.750	NR	Blood culture
Wilar 2023	Indonesia	R	EOS	176	84	92	60.40	0.869	0.870	0.928	Blood culture
Hasibuan 2024	Indonesia	R	EOS+LOS	137	48	89	11.73	0.479	0.472	0.470	Blood culture
Pamudji 2024	Indonesia	R	EOS+LOS	82	25	57	52.00	0.840	0.754	0.825	Blood culture
Zhu 2024	China	R	EOS+LOS	120	60	60	59.60	0.733	0.633	0.660	CSF/sterile cavity culture + clinical criteria
Kahvecioglu 2025	Turkey	R	LOS	60	38	22	45.24	0.816	0.636	0.787	Clinical criteria
Shen 2025	China	R	EOS+LOS	321	201	120	78.10	0.886	0.808	0.907	Clinical criteria
Yin 2025	China	R	LOS	242	142	100	57.86	0.923	0.680	0.833	Blood culture + clinical criteria

PLR = Platelet-Lymphocyte Ratio; AUC = Area Under the Curve; NR = Not Reported; R=retrospective; P=prospective; EOS= early onset sepsis; LOS=late onset sepsis.

The methodological quality of all included studies, assessed using the QUADAS-2 tool, are presented in Supplementary Table-II. All 15 studies were rated as “High risk”. In all studies, the PLR diagnostic cut-off value was determined post hoc via AUC analysis using the same dataset used for accuracy evaluation. None of the included studies pre-specified a diagnostic threshold prior to data collection.

**Supplementary Table-II:** Summary QUADAS-2 Risk of Bias and Applicability.

Author (Year)	Patient Selection	Index Test	Reference Standard	Flow & Timing	Patient Selection	Index Test	Ref. Standard	Overall
	RISK OF BIAS				APPLICABILITY CONCERNS			JUDGEMENT
Omran 2018 (Egypt)	Moderate	High	Moderate	Low	Low	Low	Moderate	High
Can 2018 (Turkey)	Moderate	High	Moderate	Low	Low	Low	Moderate	High
Arcagok 2019 (Turkey)	Moderate	High	Moderate	Low	Low	Low	Moderate	High
Nasser 2020 (Egypt)	Moderate	High	Moderate	Low	Low	Low	High	High
Zhang 2021(China)	Moderate	High	Low	Low	Low	Low	Low	High
Kurt 2022 (Turkey)	Low	High	Low	Low	Moderate	Low	Low	High
Mahmoud 2022 (Egypt)	Moderate	High	Moderate	Low	Low	Low	Moderate	High
Soliman 2022 (Egypt)	High	High	High	Low	Moderate	Low	Moderate	High
Wilar 2023 (Indonesia)	Low	High	Low	Low	Low	Low	Low	High
Hasibuan 2024 (Indonesia)	Low	High	Low	Low	Low	Low	Low	High
Pamudji 2024 (Indonesia)	Low	High	Low	Low	Low	Low	Low	High
Zhu 2024 (China)	Moderate	High	Moderate	Low	Low	Low	Moderate	High
Kahvecioglu 2025 (Turkey)	Moderate	High	Moderate	Low	Low	Low	Moderate	High
Shen 2025 (China)	Low	High	Low	Low	Low	Low	Low	High
Yin 2025 (China)	Moderate	High	Moderate	Low	Low	Low	Moderate	High

ROB = Risk of Bias; APP = Applicability Concern. Columns 2-5: Risk of Bias domains; Columns 6-8: Applicability Concern domains;

Column 9: Overall Judgement (reflects worst-rated domain). Low (green) = low risk/concern;

Moderate (orange) = moderate risk/concern (includes Unclear); High (red) = high risk/concern.

**Fig.2:**
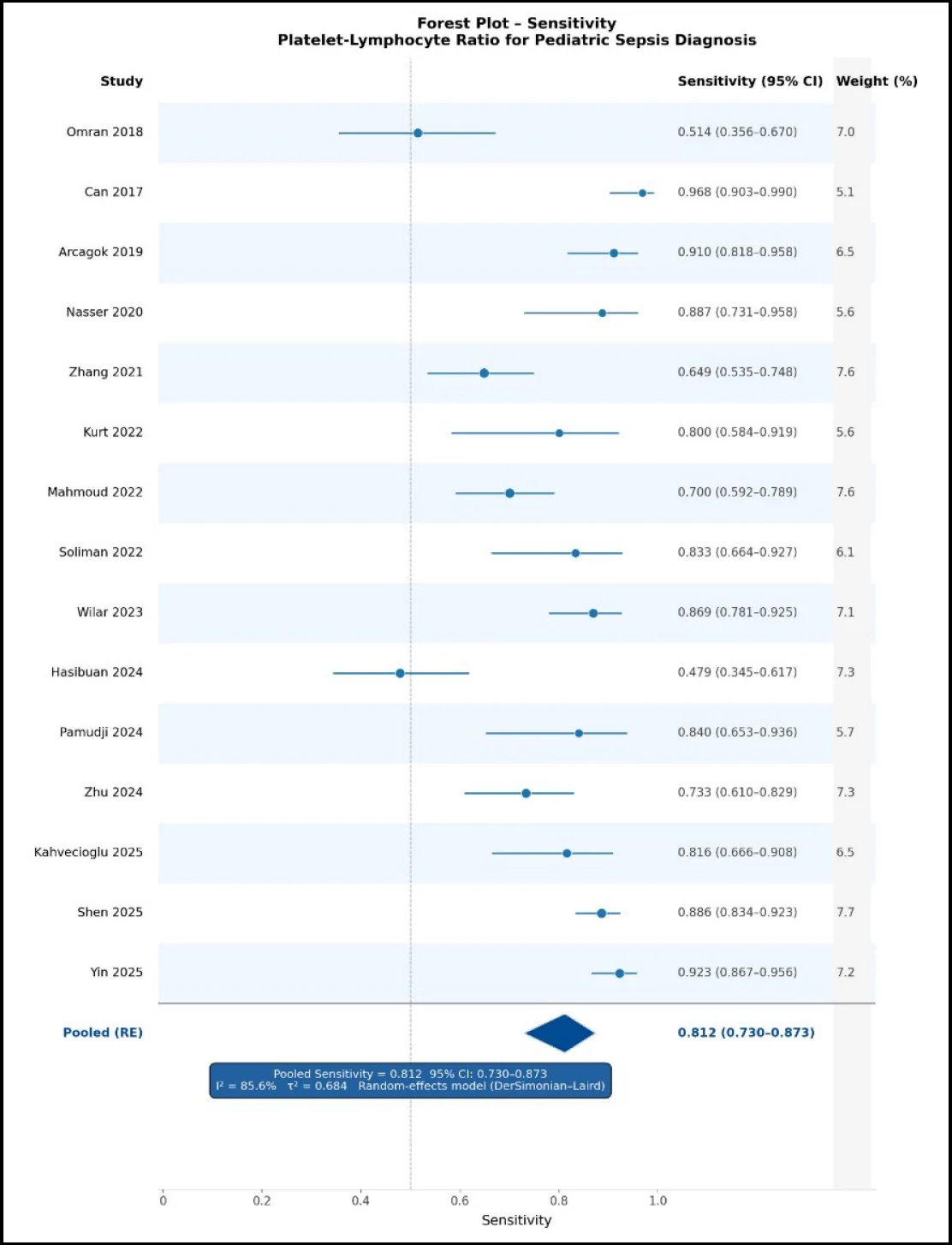
Forest plot for the sensitivity of PLR in diagnosing sepsis in pediatric patients.

**Fig.3:**
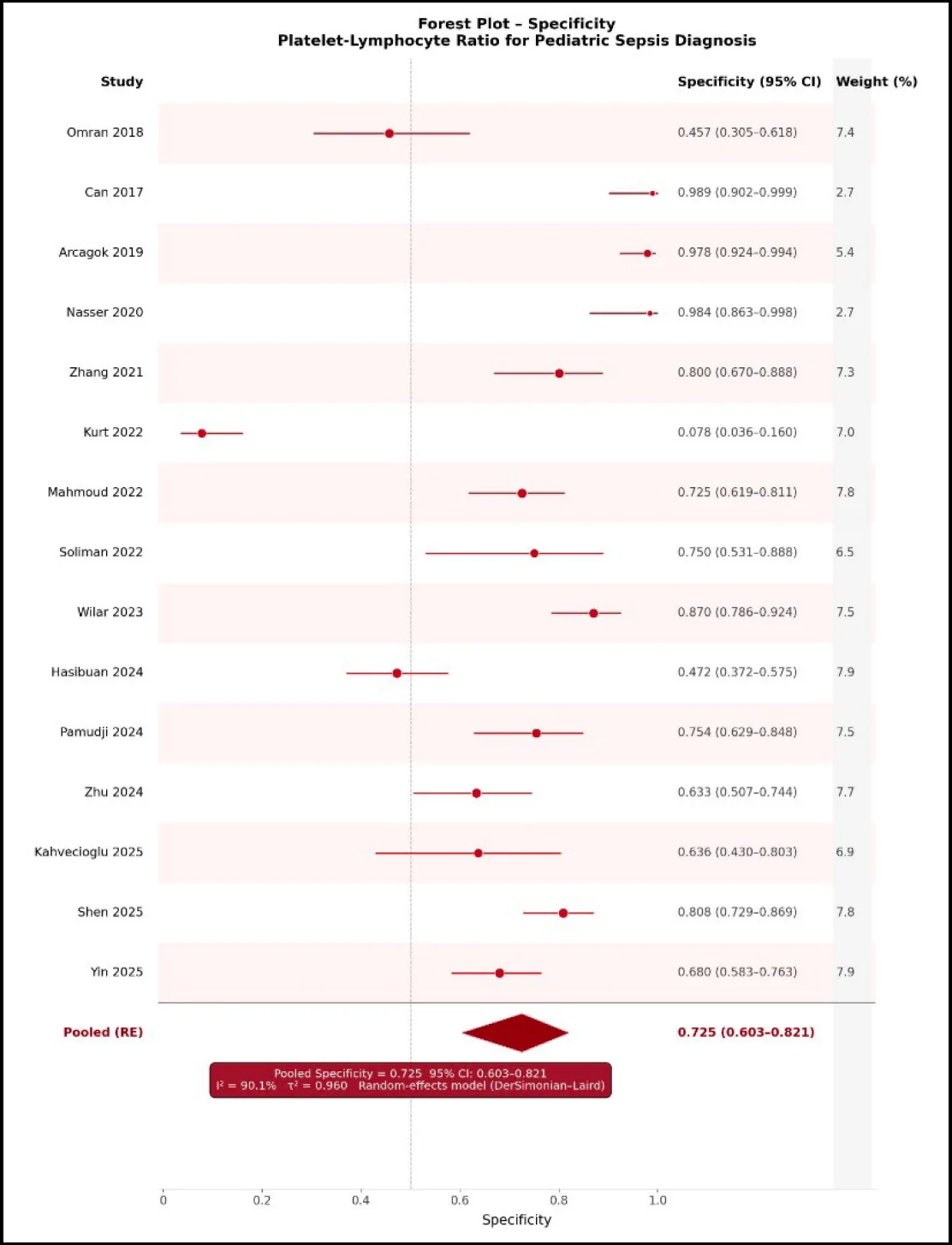
Forest plot for the specificity of PLR in diagnosing sepsis in pediatric patients.

**Fig.4:**
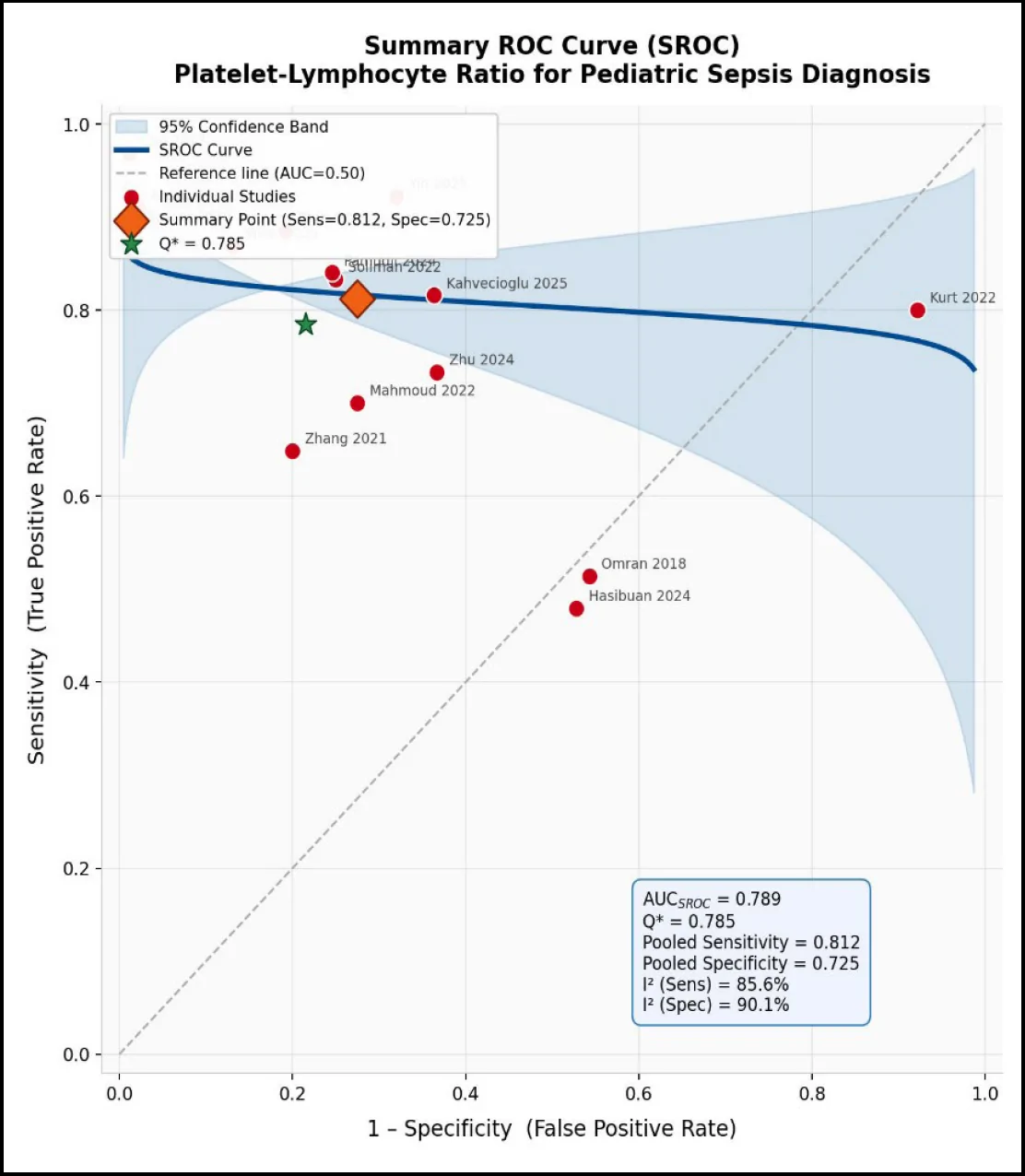
SROC curve for PLR in diagnosing sepsis in pediatric patients..

**Supplementary Fig.1:**
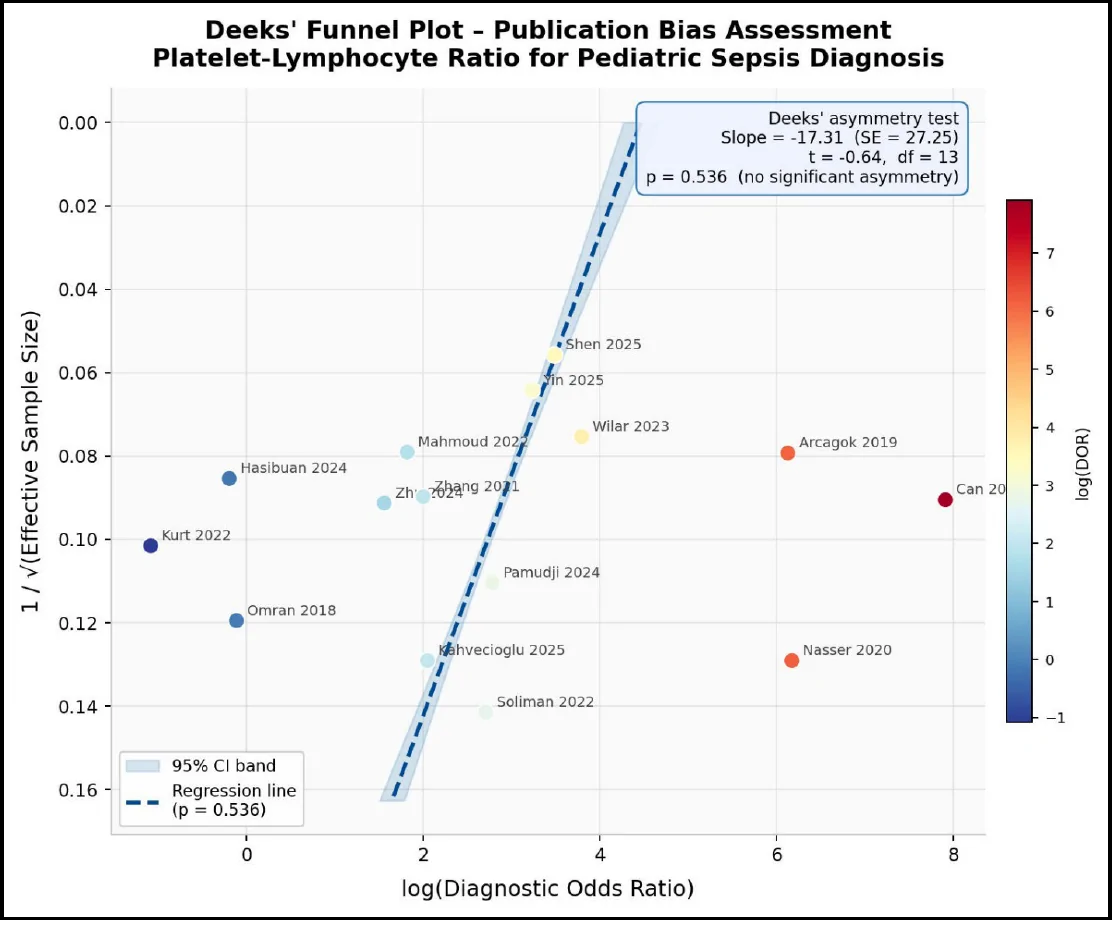
Deek’s plot for publication bias.

The pooled sensitivity of PLR for the diagnosis of pediatric sepsis was 0.812 (95% CI: 0.730–0.873), indicating that PLR correctly identified approximately 81% of septic children (Fig.2). The pooled specificity was 0.725 (95% CI: 0.603–0.821), reflecting a moderate ability to exclude non-septic individuals (Fig.3). Both estimates were associated with substantial between-study heterogeneity, as evidenced by I² values of 85.6% for sensitivity (τ² = 0.684) and 90.1% for specificity (τ² = 0.960). The summary receiver operating characteristic (SROC) curve, constructed using the Moses–Littenberg method, yielded an area AUC of 0.789, indicating moderate-to-good overall diagnostic accuracy. The Q statistic, representing the point at which sensitivity and specificity are equal on the SROC curve, was 0.785, further corroborating the moderate discriminatory performance of PLR in this population (Fig.4). Publication bias was evaluated using Deeks’ funnel plot asymmetry test (Supplementary Fig.1. The regression slope was −3.26, yielding a p-value of 0.536.

This result indicates no statistically significant funnel plot asymmetry. Subgroup Analysis results are summarized in Table-II. Heterogeneity remained substantial across all country-based subgroups for both sensitivity and specificity. Studies enrolling ≥150 patients yielded consistently superior diagnostic accuracy compared to smaller studies, with a pooled sensitivity of 0.869 (95% CI: 0.782–0.924; I² = 82.4%) and a pooled specificity of 0.823 (95% CI: 0.713–0.897; I² = 83.6%). In contrast, studies with sample sizes below 150 demonstrated lower pooled sensitivity of 0.769 (95% CI: 0.658–0.853; I² = 80.3%) and markedly lower pooled specificity of 0.650 (95% CI: 0.468–0.796; I² = 89.3%). Studies employing a PLR cut-off greater than 50 demonstrated higher pooled sensitivity of 0.840 (95% CI: 0.740–0.907; I² = 87.5%) and pooled specificity of 0.789 (95% CI: 0.683–0.866; I² = 84.1%) compared to studies using cut-off values of 50 or below (n = 6), which yielded pooled sensitivity of 0.756 (95% CI: 0.620–0.855; I² = 75.7%) and substantially lower pooled specificity of 0.589 (95% CI: 0.327–0.809; I² = 92.0%).

**Table-II:** Subgroup analysis of PLR for pediatric sepsis diagnosis.

Variable	No. of Studies	Sensitivity (95% CI)	I² (%)	Specificity (95% CI)	I² (%)
Country					
China	4	0.822 (0.666–0.915)	90.6	0.734 (0.639–0.811)	66.5
Egypt	4	0.740 (0.568–0.860)	75.9	0.717 (0.492–0.869)	78.7
Indonesia	3	0.756 (0.431–0.927)	91.3	0.722 (0.431–0.899)	93.6
Turkey	4	0.890 (0.783–0.948)	63.2	0.808 (0.182–0.988)	95.9
Sample size					
≥150 patients	5	0.869 (0.782–0.924)	82.4	0.823 (0.713–0.897)	83.6
<150 patients	10	0.769 (0.658–0.853)	80.3	0.650 (0.468–0.796)	89.3
PLR Cut-off					
>50	9	0.840 (0.740–0.907)	87.5	0.789 (0.683–0.866)	84.1
≤50	6	0.756 (0.620–0.855)	75.7	0.589 (0.327–0.809)	92.0

## DISCUSSION

Our analysis showed that PLR had a pooled sensitivity of 81%, a pooled specificity of 72.5%, and an SROC-AUC of 0.789 for diagnosing pediatric sepsis. Together, these results suggest moderate to good diagnostic accuracy, although the high heterogeneity between studies (I² >85% for sensitivity and specificity) limits confidence in the results. Additionally, no significant publication bias was found using Deeks’ funnel plot test. However, since all studies had high risk of bias, evidence must be interpreted with caution.

Previously, Bai et al.^11^ conducted a similar systematic review, but could include just six studies. In the present study, we conducted an updated literature search to expand the evidence base and improve the accuracy of diagnostic estimates. Bai et al.^11^ reported pooled sensitivity and specificity for PLR of 0.82 (95% CI: 0.63–0.92) and 0.80 (95% CI: 0.24–0.98), with an AUC of 0.87. Our estimates of sensitivity are similar (0.812), but specificity is slightly lower (0.725), with much narrower confidence intervals due to the larger and up-to-date dataset. Notably, a very wide CI was observed for specificity in the study by Bai et al.^11^ (0.24–0.98), indicating the imprecision of the estimate, which was addressed in our meta-analysis. The AUC in our analysis (0.789) is slightly lower than the previous value of 0.87, likely due to the inclusion of nine additional studies, which have refined the value rather than suggesting a decline in the effectiveness of PLR.

Benchmarking PLR with other haematological indices from the complete blood count offers useful insights. The most studied comparator is the neutrophil-to-lymphocyte ratio (NLR). A large meta-analysis of 30 studies by Chen et al.^29^ reported pooled sensitivity and specificity for NLR in neonatal sepsis of 79% (95% CI: 62–90%) and 91% (95% CI: 73–97%), respectively. Bai et al.^11^ also found NLR sensitivity and specificity of 0.76 and 0.82, with an AUC of 0.86. Overall, these findings show that NLR offers better specificity than PLR. Conversely, PLR often shows slightly higher or comparable sensitivity, meaning it may identify fewer septic neonates but with fewer false positives. Interestingly, Bai et al.^11^ reported nearly identical AUCs for both markers (0.87 vs. 0.87), indicating they might be interchangeable at the summary level, with differences mainly in the sensitivity-specificity balance. Notably, only platelet parameters have been used to diagnose sepsis in children. A meta-analysis shows that mean platelet volume has fair diagnostic accuracy for neonatal sepsis, with sensitivity and specificity of 0.675 (95% CI: 0.536–0.790) and 0.733 (95% CI: 0.589–0.840), respectively.^30^

Understanding the significance of PLR involves comparing it with the most common biochemical markers. A recent network meta-analysis by Huang et al.,^10^ which assessed 20 haematological parameters across 311 studies, identified presepsis as one of the most sensitive and specific markers. Although PLR was not part of their analysis, presepsis ranked higher than CRP, PCT, NLR, and platelet counts. Ruan et al.^31^ performed a meta-analysis showing pooled sensitivities of 0.71 for CRP and 0.85 for PCT in neonatal sepsis, with AUCs of 0.85 and 0.91, respectively, and an AUC of 0.96 when combining PCT and CRP. This demonstrates a significant diagnostic benefit of PCT and its combination with CRP over PLR alone, in terms of overall accuracy, though these methods are more expensive and logistically demanding, especially in resource-limited settings lacking specialised immunoassay platforms. Conversely, PLR, derived from a routine complete blood count that can be obtained within hours at minimal additional cost, plays a unique and complementary role. Its sensitivity of 0.812 compares favourably to CRP’s 0.71, but is lower than PCT’s 0.85. Clinically, PLR can serve as a rapid, inexpensive triage tool when advanced biochemical markers are unavailable or as an adjunct to improve sensitivity when used alongside CRP or PCT.

A major limitation of this analysis was the high heterogeneity between studies which warrants caution in the interpretation of the results. A subgroup analysis was performed based on different parameters, but heterogeneity remained, indicating that other factors such as demographics, disease severity, and comorbidities might have influenced the results. Despite this, the subgroup analysis revealed some notable findings. Studies with larger sample sizes (≥150 patients) consistently showed better diagnostic accuracy than smaller studies. This suggests that PLR performance estimates in small, single-centre studies could be high due to overfitting and spectrum bias. We also noted that cut-offs above 50 were associated with higher specificity (0.789 vs. 0.589 for cut-offs ≤50). The wide variation in cut-off values across studies (from 0.02 to 94.05) likely contributes heavily to the heterogeneity and prevents a universal diagnostic threshold from being recommended for this biomarker.

### Limitations of the study:

First, all 15 included studies were deemed high risk of bias according to QUADAS-2. This was because the PLR cut-off values were derived post-hoc from the same dataset in all studies to assess diagnostic accuracy. This technique likely inflates sensitivity and specificity estimates and thereby restricts the validity of the pooled results. Second, the evidence is available only from four countries. There was no data from regions that carry the highest global burden of neonatal sepsis, like sub-Saharan Africa or South Asia thereby limiting the generalizability of these findings to the populations in greatest need. Third, most studies focused on neonatal populations. This restricts our ability to draw conclusions for older pediatric groups, where the disease processes and PLR reference ranges may differ. Fourth, the threshold values for PLR varied considerably among studies. It shows the lack of a standardised cut-off in the field, and adds to the heterogeneity of the overall results. Finally, there were variations in reference standards in the studies with some using of both clinical and microbiological criteria which may have further contributed to heterogeneity in the analysis.

Despite some limitations, PLR offers practical benefits as a point-of-care tool, especially in resource-limited pediatric and neonatal settings where PCT testing or advanced biomarkers might not be available. It can also support nurses with early disease assessment, monitoring vital signs, preserving organ function, managing fluids, controlling infections, and providing pain, psychological, and nutritional care. Additionally, PLR may help standardize sepsis nursing procedures, improve nurses’ ability to anticipate clinical changes, and strengthen emergency response skills.

## CONCLUSION

Evidence from studies with high-risk of bias indicates that PLR may have moderate-to-good diagnostic accuracy for pediatric sepsis, with a pooled sensitivity of 0.812, specificity of 0.725, and an SROC-AUC of 0.789. However, the presence of a high risk of bias across all included studies and variable PLR cut-offs in the literature suggests that these estimates should be interpreted cautiously until future prospective studies with predefined cut-offs are conducted. Moreover, since most studies were on neonatal population, evidence may not be generalized to older pediatric populations.

### Recommendations

Future studies should explore on older pediatric populations to provide better evidence for this cohort. Also, studies should assess if targeted nursing interventions can lead to earlier diagnoses, reduce complications like organ failure or worsening infections, shorten hospital stays, increase treatment success and nursing satisfaction, and serve as a reference for consistent sepsis care. Researchers should also consider multi-center prospective studies that establish a PLR threshold beforehand to avoid bias, as seen in current literature. Comparing PLR with NLR and SII in well-powered prospective cohorts, stratified by early versus late sepsis and age groups would help clarify how these markers relate to each other.
